# SOCIAL INTERACTION AND DEPRESSIVE SYMPTOMS: A COMPARISON OF MID- TO LATER-LIFE SAME- AND DIFFERENT-SEX COUPLES

**DOI:** 10.1093/geroni/igae098.0526

**Published:** 2024-12-31

**Authors:** Sara Mernitz, Michael Garcia, Debra Umberson

**Affiliations:** The University of Texas Austin, Austin, Texas, United States; The University of Texas Austin, Austin, Texas, United States; The University of Texas Austin, Austin, Texas, United States

## Abstract

Social interaction with family and friends plays a crucial role for individual mental health with notable gendered patterns. Yet, previous studies fall short in explaining how the frequency of social interactions change over time for women and men in same-sex and different-sex married couples and how such change may influence depressive symptoms from midlife to late adulthood. Using dyadic longitudinal data from the Health and Relationships Projects (HARP), we examine patterns of change in social interaction frequency over a 6-year period for mid-to-later life U.S. women and men in same-sex and different-sex marriages (n = 616 individuals; 320 couples) and their subsequent associations with depressive symptoms. We use mixed-effects multilevel modeling and find that the frequency of social interaction with family members decreased over a period of 6 years for same-sex couples (i.e. women married to women; men married to men), but not different-sex couples. Frequency of social interaction with friends also decreased, but only for men married to men. Decreased social interaction with family members was associated with increased depressive symptoms for women married to women, but not men married to men or different-sex couples; decreased social interaction with friends was associated with increased depressive symptoms for men married to men only. Taken together, these findings illustrate how change in social interaction frequency influences mental health within marriage from mid-to-later life and underscores the importance of including same-sex couples when considering both gender differences in such linkages.

